# Hereditary cancer genes are highly susceptible to splicing mutations

**DOI:** 10.1371/journal.pgen.1007231

**Published:** 2018-03-05

**Authors:** Christy L. Rhine, Kamil J. Cygan, Rachel Soemedi, Samantha Maguire, Michael F. Murray, Sean F. Monaghan, William G. Fairbrother

**Affiliations:** 1 Molecular and Cellular Biology and Biochemistry, Brown University, Providence, Rhode Island, United States of America; 2 Center for Computational Molecular Biology, Brown University, Providence, Rhode Island, United States of America; 3 College Hill Research, Barrington, Rhode Island, United States of America; 4 Geisinger Health System, Danville, Pennsylvania, United States of America; 5 Division of Surgical Research, Department of Surgery, Alpert School of Medicine at Brown University and Rhode Island Hospital, Providence, Rhode Island, United States of America; 6 Hassenfeld Child Health Innovation Institute of Brown University, Providence, Rhode Island, United States of America; Oslo University Hospital, Norwegian Radium Hospital, NORWAY

## Abstract

Substitutions that disrupt pre-mRNA splicing are a common cause of genetic disease. On average, 13.4% of all hereditary disease alleles are classified as splicing mutations mapping to the canonical 5′ and 3′ splice sites. However, splicing mutations present in exons and deeper intronic positions are vastly underreported. A recent re-analysis of coding mutations in exon 10 of the Lynch Syndrome gene, *MLH1*, revealed an extremely high rate (77%) of mutations that lead to defective splicing. This finding is confirmed by extending the sampling to five other exons in the *MLH1* gene. Further analysis suggests a more general phenomenon of defective splicing driving Lynch Syndrome. Of the 36 mutations tested, 11 disrupted splicing. Furthermore, analyzing past reports suggest that *MLH1* mutations in canonical splice sites also occupy a much higher fraction (36%) of total mutations than expected. When performing a comprehensive analysis of splicing mutations in human disease genes, we found that three main causal genes of Lynch Syndrome, *MLH1*, *MSH2*, and *PMS2*, belonged to a class of 86 disease genes which are enriched for splicing mutations. Other cancer genes were also enriched in the 86 susceptible genes. The enrichment of splicing mutations in hereditary cancers strongly argues for additional priority in interpreting clinical sequencing data in relation to cancer and splicing.

## Author summary

To understand the extent to which disrupted pre-mRNA splicing causes human disease, we re-analyzed coding mutations in *MLH1*, one of the causal genes of Lynch Syndrome. We found that a high fraction of the *MLH1* coding mutations resulted in disrupted splicing. To further investigate a more general role of defective splicing across human disease genes, simulation strategies were used to identify 86 disease genes prone to splice site mutations. In these 86 genes, there was an enrichment of cancer genes including the three main casual genes of Lynch Syndrome (*MLH1*, *MSH2*, and *PMS2*). Thus, it appears defective splicing may be the main driver of Lynch Syndrome and other cancers. Genes prone to splicing mutations have certain features that allow for the comprehensive prediction of splicing-prone diseases genes in the human genome. Our findings strongly argue for additional clinical sequencing prioritization in both cancer genes and genes prone to splice site mutations.

## Introduction

As the cost of sequencing technologies is declining, the number of genomes and exomes sequenced is increasing, resulting in an expanding archive of genetic variation in both diseased and healthy individuals [1, 2]. To keep pace with the ever growing variant archive, *in silico* tools are being created to determine the functional impact of variants discovered [3–6]. However, most tools used to determine the pathogenicity of variants rely on in *silico* methods aimed at deciphering protein features associated with the variant and fail to take into account the potential regulatory functions of sequences in gene processing mechanisms and expression [7].

The sequences that encode for proteins (exons) and the intervening, noncoding sequences (introns) are known to have an important regulatory role in an RNA processing mechanism known as precursor messenger RNA (pre-mRNA) splicing. Variants that alter the regulatory regions necessary for splicing typically result in the deletion of large portions of the coding sequence and generally result in a non-functional protein [8]. Among the reported sequence variants, splicing mutations located at the 5′ and 3′ canonical exon-intron boundaries, or splice sites, make up 13.4% of the disease-causing mutations reported in the Human Gene Mutation Database (HGMD) [9]. However, in addition to splicing variants located at the splice sites, splicing variants within the exonic sequences can also modulate splicing by altering the multitude of exonic splicing enhancers (ESE) and silencers (ESS) present in exons. Due to the difficulty in classifying exonic mutations as splicing mutations, it is becoming evident that new methods and tools will need to be implemented to correctly and thoroughly identify exonic splicing mutations (ESM). An ESM is a hereditary disease allele that falls within the exon and was originally annotated as a protein coding mutation. For the purpose of this analysis, a splice site mutation (SSM) falls within the 5`splice site (i.e. -3 to +6 position 5`end of the intron) or the 3`splice site (i.e. -20 to +3 position of the 3`end of the intron). Recently, studies have been aimed at re-analyzing reported sequence variants for splicing defects [10, 11]. Much of this work suggests that splice-altering variants are more common than previously anticipated. For example, a recent re-analysis of 20 coding mutations located in exon 10 of *MLH1*, reveal a high proportion of previously uncharacterized ESM (17 of the 20 or 77%) [11]. In fact, using the position dependence of splicing elements as a measure to infer disruptive splicing, it has recently been predicted that one-third of all disease-causing variants lead to aberrant splicing [12].

Here, we present a comprehensive analysis of splicing mutations in human disease. We report 86 genes enriched for SSM, in patients that present with hereditary disease (see **Materials and Methods**). Of these 86 SSM-prone genes, three were the main causal genes of Lynch Syndrome (*MLH1*, *MSH2*, and *PMS2*), which account for 32%, 39%, and 14% of Lynch Syndrome cases, respectively [13]. Lynch Syndrome, a cancer-susceptibility disorder caused by autosomal dominant germline mutations in the mismatch repair (MMR) genes above, accounts for ~5% of all colorectal cancers. In addition, individuals with Lynch Syndrome have an elevated risk of developing early-onset colorectal and endometrial cancers [14]. With colorectal cancer being the second leading cause of cancer death in the United States [15], it will be imperative to understand the disease mutational mechanisms underlying Lynch Syndrome to aid in the development of therapeutic strategies.

However, not only were Lynch Syndrome genes members of the 86 SSM-prone genes, but it was also found that the COSMIC set of cancer genes were overrepresented [16]. This work highlights the importance of allocating additional priority to investigating splicing defects in a described set of genes, many of which have been associated with some feature of cancer risk or progression.

## Results

### *MLH1* has a high proportion of splicing mutations that are non-uniformly distributed among its exons

**Fig 1:**
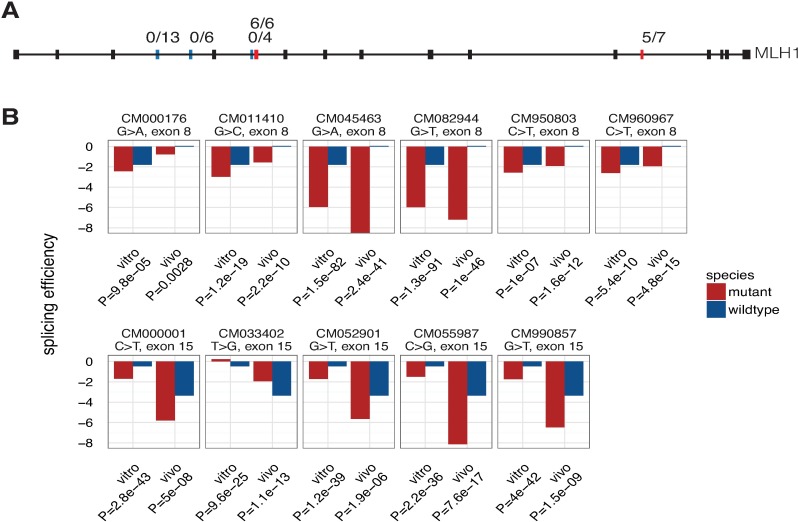
*MLH1* is frequently disrupted by splicing mutations. **A.** Disease coding mutations in exons 4, 5, 7, 8 and 15 of *MLH1* were analyzed with MaPSy. While none of the mutations in exons 4, 5 and 7 (blue bars) were found to disrupt splicing, almost all of the mutations tested in exons 8 and 15 (red bars) significantly altered splicing (100% and 71%, respectively). **B.** Splicing efficiency of wildtype (blue) and mutant (red) alleles that were tested with MaPSy in exons 8 and 15 of *MLH1*.

A recent analysis of coding mutations located in exon 10 of *MLH1* revealed a high level of coding mutations (17/22 or 77%) altered the splicing of exon 10 [11]. To see if the results of this survey of *MLH1* exon 10 was indicative of high levels of splicing phenotypes in exonic mutations across all genes, a larger pool of exonic variants (outside canonical splice sites) was analyzed using a high-throughput reporter assay, MaPSy [10]. MaPSy was used to screen variants in five additional *MLH1* exons. Of the 36 pathogenic *MLH1* exonic mutations surveyed with MaPSy, 11 (30.5%) affected splicing (**Fig 1A and 1B, S1 Table**) in an *in vivo* minigene assay and in an *in vitro* splicing assay. On average, disease causing point mutations disrupt splicing 10% of the time (MaPSy 5K panel, *n* = 4,964 alleles) [10]. In other words, the rate of splicing misregulation in *MLH1* disease alleles was almost three times higher than the background rate of splicing disruption in disease alleles. Mapping potential exonic splicing regulatory sequences (ESRs) [17] in the *MLH1* exons analyzed in MaPSy revealed exon mutations that altered splicing resulted in a greater difference in wild type (wt)–mutant (mt) ESR scores than mutations not resulting in a splicing defects (average ∆ESR score 1.845 and 0.8583 respectively, *P* = 0.0280 Mann-Whitney, **S1 Fig, S1 Table**). *MLH1* missense and nonsense mutations were found to frequently disrupt splicing *in vitro* and *in vivo*: 6/22 (27%) missense mutations and 5/14 (36%) nonsense mutations. Taken together, this data a) confirms the previous report that exonic mutations in *MLH1* frequently disrupt splicing b) exonic mutations that alter ESR signals are more likely to result in a splicing defect, and c) suggests that the rate of splicing disruption is not homogenous across genes (i.e. *MLH1* is an outlier).

Interestingly, ESMs were also disproportionately distributed among the exons within the *MLH1* gene. Of the five exons that were included in this study, three had no ESMs. However, all the exonic mutations in exon 8 (6/6) and 71% (5/7) of the mutations in exon 15 significantly altered splicing (**Fig 1A and 1B**). Thus, it appears that certain exons in *MLH1* are more prone to splicing disruption. To investigate the possibility that certain exons may be more prone to ESMs, a permutation approach was used to identify exons that exceeded the expected number of ESMs discovered (see **Materials and Methods**). 11 of the 2,061 exons analyzed using MaPSy were predicted with a *P* < 0.01 to have more ESM than expected (**S2 Fig**). Remarkably, two of these 11 exons identified in the simulation as being enriched for ESMs were *MLH1* exon 8 and exon 15, further confirming the previous finding.

**Fig 2:**
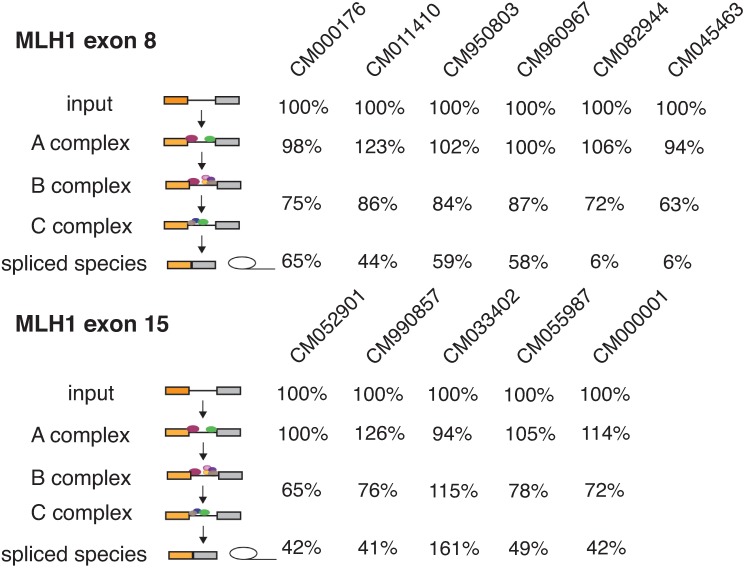
*MLH1* ESM affect different stages of spliceosome assembly. The percentages of mutant mRNA retained in each stage of the assembly relative to wildtype mRNA are shown for all ESM that were identified in *MLH1* exon 8 and 15. The majority of ESM were blocked in the transition from A and B complex. Two of the ESM (CM082944 and CM04546) in exon 8 also slowed down the final transesterification reactions to yield spliced mRNA and the lariat.

To mechanistically investigate the defective splicing of *MLH1* mutations, the representation of *MLH1* alleles in the fractions of the *in vitro* spliceosomal assembly assay was examined (see **Materials and Methods** and **S3 Fig**). Here, the accumulation of an allele in intermediate complexes was interpreted as an indication that the allele blocked the next stage of spliceosome assembly [10]. In general splice site recognition is thought to occur early in spliceosome assembly [8, 18], however for the ESMs in *MLH1*, the disruption occurred later. 63% of exonic splicing mutations were primarily blocked at the A complex in transition to the B complex and 37% were blocked at the B complex (**Fig 2**). Several mutants reduce more than one step in the assembly (**Fig 2**). As expected, adjacent mutations that were close enough to fall within the same *cis*-element shared a similar pattern of disruption. In effect, these clusters of variants mutationally defined a particular cis-elements required for particular spliceosomal transitions (e.g. **Fig 2**, CM045463 and CM082944).

### Non-uniform distribution of SSM across disease genes

**Fig 3:**
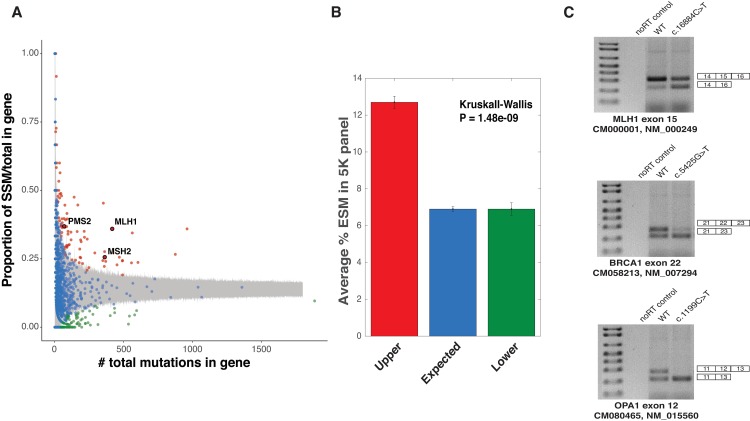
Non-uniform distribution of splicing mutations across disease genes. **A.** SSM versus all exonic mutations in the HGMD with regions of 99.9% confidence interval shown in gray. Genes with more, expected, and less SSM are shown in red (Upper), blue (Expected), and green (Lower), respectively. Location of *MLH1*, *MSH2*, and *PMS2* are highlighted and labeled. **B.** Percent ESM of total mutations tested using MaPSy in each category. **C**. Due to the inability of MaPSy to observe mutant-specific exon skipping events (as a result of the identical flanking exons), ESMs found in MLH1, BRCA1, and OPA1 were validated as individual wildtype and mutant minigene constructs. All three mutant constructs showed exon skipping events, which were not shown in wildtype constructs.

The surprisingly high fraction of disease-causing splicing mutations both reported in the splice-sites and unreported in exonic positions of *MLH1* (as shown by the MaPSy 5K panel) may be due to chance or the enrichment for splicing mutations in the gene/disease. To eliminate the null hypothesis, Monte Carlo (MC) simulations were used to generate a distribution of SSM frequencies for each gene given the total number of mutations reported in that gene (see **Materials and Methods**). Of the ~3,600 disease genes reported in the HGMD, 86 genes, including the three main casual Lynch Syndrome genes (*MLH1*, *MSH2*, and *PMS2*), had more SSM than expected based on the distribution of SSM in the HGMD dataset (**Fig 3A, S2 Table**).

Although SSM generally have a severe impact on splicing outcome by disrupting the essential interactions with the core spliceosome components, variants located within the exonic sequence can also alter splicing by disrupting the myriad of exonic splicing regulatory (ESR) elements [18]. Using the results obtained from the MaPSy 5K panel, we found that the 86 SSM-prone genes not only had a higher proportion of mutations in the canonical splice sites but also contained exonic mutations that were almost twice as likely to disrupt splicing as exonic mutations that occurred in the remaining genes (1.84-fold effect; *P* = 1.48 x 10^−9^, Kruskal-Wallis, **Fig 3B**). These results suggest that the 86 SSM-prone genes are not only prone to SSMs but also to ESMs, with three ESMs in the 86 SSM-prone genes being validated in individual minigene constructs (**Fig 3C**).

### Cancer genes are enriched in SSM–prone genes

**Fig 4:**
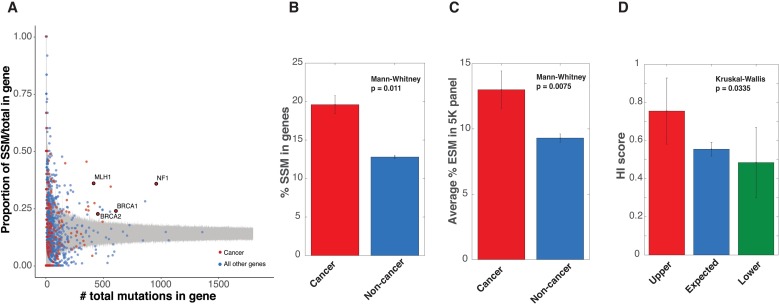
Enrichment of cancer genes in SSM-prone genes. **A.** SSM versus all exonic mutations in the HGMD with regions of 99.9% confidence interval shown in gray. COSMIC cancer genes are highlighted in Red. *MLH1*, *BRCA1*, *BRCA2*, and *NF1* are highlighted and labeled. **B-C.** Average percent of SSM or ESM in cancer genes versus non-cancer genes reported in HGMD. **D.** Average HI score of cancer genes in Upper, Expected, and Lower categories of genes.

We next sought to determine if a certain class of disease genes were overrepresented in the 86 SSM-prone genes (**S2 Table**). The initial report of an association between *MLH1* and splicing mutations also associated other cancer related genes such as *BRCA1*, *BRCA2*, and *NF1* with disrupted splicing. Furthermore, Gene Ontology (GO) enrichment analysis [19] of the 86 SSM-prone genes revealed an enrichment of genes associated with the DNA repair pathway (*P* = 2.53x10^-2^, **S3 Table**), a pathway commonly associated with cancer phenotypes [20, 21]. To determine if cancer genes were overrepresented in the 86 SSM-prone genes, the Catalogue of Somatic Mutations in Cancer (COSMIC) was crossed referenced with the HGMD disease genes [16]. Of the 609 cancer genes associated with elevated somatic mutations in tumors (i.e. the COSMIC gene set), 280 were reported with germline mutations in hereditary cancers (i.e. HGMD). These cancer genes were particularly enriched in the SSM-prone genes (1.5 fold in the upper category 20/86, *P* < 0.01, permutation test, **Fig 4A**). Not only were cancer genes overrepresented in the SSM-prone genes, but they also contained 1.5-fold more SSM and 1.4-fold more ESM than the rest of the genes in the HGMD (*P* = 0.011 and *P* = 0.0075, Mann-Whitney, for SSM and ESM respectively, **Fig 4B and 4C**). When further dividing the cancer genes into oncogenes and tumor suppressor genes (TSG), it became apparent that TSG have more SSM and ESM than the rest of the genes in the HGMD (*P* = 0.0178 and *P* = 1.14 x 10^−4^, Mann-Whitney, for SSM and ESM respectively, **S4 Fig**). However, this enrichment for SSM and ESM was not apparent when comparing oncogenes to the rest of the genes in the HGMD (*P* = 0.4821 and *P* = 0.1914, Mann-Whitney, for SSM and ESM respectively, **S4 Fig**). Thus, it appears that TSG are more prone to splicing dysfunction most likely due to their loss-of-function disease mutational mechanism.

### Several features modulate the sensitivity of genes to SSM

A number of genomic and sequence features have been implicated in the context of splicing [17, 22–25]. We, therefore, sought to determine if genomic and sequence features existed that would result in the predisposition of a gene to SSM. In fact, multiple features appeared to modulate the predisposition of a gene to SSM. When analyzing 19 genomic features (**S4 Table**) [17, 23, 26–28], we found that the 86 SSM-prone genes contained 2.5 fold more introns than the rest of the genes in the analysis (*P* = 2.54 x 10^−14^, Kruskal-Wallis, **S5 Fig**). Thus a trivial explanation for predisposition of the 86 SSM-prone genes is the larger mutational target presented by their higher number of splice sites. To determine if the SSM-prone genes were predisposed due to the number of introns, we repeated the MC simulation normalizing for the number of introns (see **Materials and Methods**). Surprisingly, this correction did not dramatically alter the result. After normalization, about 74.4% (64/86) of the genes that were significantly enriched for splice site mutations, were present in the recalculated SSM-prone gene list (**S5 Table**).

**Fig 5:**
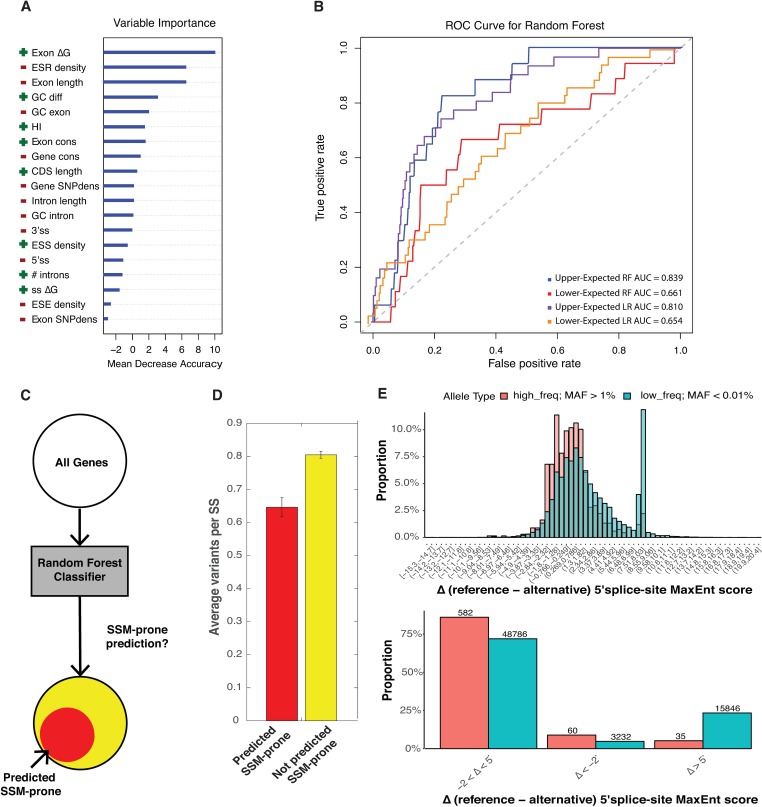
Random forest classification and prediction of SSM-prone genes. **A.** The order of variable importance by mean decease in accuracy for SSM-prone genes versus genes with an expected number of SSM. The directions that associate with SSM-prone genes are indicated, positive directions are green, and negative directions are red. **B.** Classification performance of the random forest models and the logistic regression models was calculated as the area under the curve (AUC) in receiver operating characteristic (ROC) analysis. **C.** Scheme of random forest classification on all genomic genes. **D.** Average proportion of low frequency ExAC splice-site variants per splice-site in predicted SSM-prone genes (probability: 0.60–0.86) versus genes not predicted to be SSM-prone (*P* = 6.1043e-18, Mann-Whitney). **E.** Common variants are depleted from the category of variants that cause loss of splice-site signal at the 5′ splice-site (upper plot). Rare variants are enriched in the range of the splice site signal scores that abolish 5′ splice-site recognition (lower plot).

In addition to having more introns, the 86 SSM-prone genes are generally more haploinsufficient (HI), have shorter and more structured exons (predicted to have more base-pairing interactions), and less conserved variants found in the exomes of ~60,000 healthy individuals [26] (**S5 Fig**). To determine the relative contribution of each feature to the classification, several machine learning approaches were trained on the HGMD mutation dataset. Briefly, the Random Forest (RF) [29] and a Logistic Regression (LR) predictive models were utilized to predict whether a gene would be associated with a significant excess of SSM (red dots, **Fig 3A**; for feature ranking please see **Materials and Methods**). The model indicates that HI genes and genes with less structured exons have a higher risk of being frequently affected by SSM (**Fig 5A**). In addition to feature prioritization, the classifier was also used to predict additional genes that may be prone to SSM but had not yet been identified as human disease genes. To test the performance of both classifiers, ROC curve analysis was performed. The mean area under the curve was measured for both machine learning models. The RF model was the most predictive (AUC = 0.839, **Fig 5B,** see **S6 Table** for cross-validation). A control classifier trained to predict genes that were not prone to SSM (i.e. Lower-Expected genes, **Fig 3A**, green) was considerably less accurate, presumably because this category is lower confidence with fewer associated mutations overall.

As haploinsufficiency was an important feature in the prediction of SSM predisposition (upper category) and splicing defects generally result in a severe loss of gene function, it is possible that the degree of haploinsufficiency largely determines a genes predisposition to SSM. However, the RF model still performed well with HI removed (AUC = 0.805). Therefore, it does not appear that there is a single dominant feature such as HI or the number of introns that drives the accuracy of the predictor. Instead it is most likely a combination of features that determine a genes predisposition to SSM. This analysis suggests that the prediction of genes predisposed to SSM using a broad spectrum of features is feasible and could potentially be used to identify new disease genes that are prone to splicing mutations.

### 499 novel genes predicted to be prone to SSM

In order to identify new disease genes that are prone to splicing mutations, the predictive model was applied to ~13,000 non-disease associated genes (**Fig 5C**). While the classifier was run at a range of stringencies. Using a probability cutoff of 0.6–0.86 returned by the classifier, 499 genes were predicted to be SSM-prone (see **Materials and Methods, S7 Table**). It is possible that these 499 genes were not previously identified as disease genes because their function was required for organismal viability. To explore the degree to which variation can be tolerated in these 499 genes, the aggregated exome sequencing data from 60,706 presumably healthy individuals provided by Exome Aggregation Consortium (ExAC) [26] was cross referenced with the 499 genes. The 499 predicted SSM-prone genes had significantly fewer reported ExAC splice site (SS) region variants than the rest of the testable genomic genes in the analysis (**Fig 5D,** *P* = 6.1043e-18, Mann-Whitney). This analysis suggests that the splicing elements in the predicted SSM-prone genes are evolving under a higher level of selective pressure. However, this analysis considers all variations equivalently making no distinction between neutral variants and clear loss of function variants. For the variants that fall within the splice sites, position weight matrix (PWM) models can be used to evaluate whether a variant represents a stronger or weaker match to the splice site consensus. In other words, PWM can potentially distinguish loss of function splicing mutants from neutral variation. In this analysis, variants that greatly weaken the match to a splice site model (e.g. ∆ > 5, **Fig 5E**) and would be expected to result in a loss of function are four fold underrepresented in common single nucleotide polymorphisms (SNPs). This suggests a scenario where loss of function variants are eliminated from the variant pool before the SNP can reach a reasonable frequency in the population. Conversely, variants that fall within the 5′ ss but strengthen the agreement of the site to the consensus tend to accumulate in the high frequency set (e.g. ∆ < -2, **Fig 5E**.). The same trend is observed in variants that localize to the 3′ ss (**S6 Fig**). An independent measure of selection can be found in analysis that maps obvious loss-of function variants to the predicted SSM-prone genes. For example, 3,230 genes that were depleted of predicted protein-truncating variants (PTV’s) in the exomes of 60,706 individuals are a gold standard for genes in which loss of function variants are poorly tolerated [26]. While PTV depletion is unrelated to splicing, there is a four or five-fold enrichment of predicted SSM-prone genes in this dataset (**S7 Fig**, *P* = 7.53e-98 Fisher’s Exact, **S7 Table**).

The lower proportion of ExAC variants located in the genomic genes predicted to be SSM-prone and the enrichment of PTV-intolerant genes in the SSM-prone genes suggests that they are intolerant to variation and appear to be functionally important genes. It is therefore more likely that splice disrupting variants that map to these genes will be deleterious. To gain more insight into the uncharacterized set of predicted SSM-prone genes, GO Enrichment analysis was performed. Regulation of cell cycle (*P =* 2.20e-2) and mitosis (*P =* 5.08e-5) were the two functions enriched in predicted SSM-prone genes (**S8 Table,** for individual GO term associations see **S7 Table**). Since the hallmark of cancer is generally the abnormal growth and division of cells, it is possible that mutations within this set may play some yet undiscovered role in cancer. While a more complete characterization of these genes awaits future study, an online browser has been developed to visualize the splicing results of the exonic mutations assessed in the SSM-prone cancer genes studied using MaPSy (**S9 Table**).

## Discussion

High rates of splicing disruption were reported in the literature for exonic variations in a panel of exons in medically important genes [10, 11, 30, 31]. As there have been a wide variety of estimates of the degree to which splicing defects accompany disease-causing mutations, this current study was initially intended to perform this analysis at a larger scale. The query was expanded to include both exonic and splice site mutations in the set of human genes known to cause hereditary disease. This analysis confirmed the initial reports of high mutation rates in the genes studied but also demonstrated that the degree to which splicing causes disease varies significantly from gene to gene.

Recent analysis of mutations in *MLH1*, a mismatch repair gene tied to Lynch Syndrome, indicated a high degree of splicing disruption as a common disease mechanism of exon 10. Due to Lynch Syndrome’s highly penetrant nature in inherited colorectal cancer predisposition, understanding the pathogenesis of the syndrome will be fundamental in devising treatment methods. To further analyze the disease mechanisms in *MLH1*, 36 additional exonic mutations were tested with 31% disrupting splicing (**Fig 1**). The degree to which exonic mutations affect splicing also vary across exons. For example, in *MLH1*, all of the ESM occurred in two of the five exons tested (**Fig 1A**). Earlier work on spliceosome assembly suggested a mechanism where the spliceosome ‘commit’ to splice sites early in the process [32]. In contrast, many of these mutations that disrupted splicing fairly late in the assembly of the spliceosome (**Fig 2**). Overall, the MaPSy assay demonstrated a three-fold increase in likelihood that a missense mutation in *MLH1* would result in a splicing defect. This study confirms earlier findings of high frequency of splicing defects in *MLH1* mutants, but also suggests that the Lynch Syndrome genes, *MLH1*, *MSH2* and *PMS2*, and the other tested genes are outliers and are prone to splicing disruption.

A major conclusion drawn from this study is the existence of a class of diseases that are often caused by splicing mutations (i.e. SSM and ESM). The role that splicing defects plays in genetic disease varies across disease genes but genes with elevated SSM also have elevated ESMs (**Fig 3**). The discovery of a class of genes prone to splicing mutations, led to an exploration of what features and cellular functions that predisposed splicing genes encode. GO term analysis indicated that many of these genes were involved in cancer initiation and progression. Defining a set of ‘cancer’ genes at the intersection of the COSMIC and HGMD dataset revealed a significant elevation of SSM and ESM in cancer genes, including genes involved in Lynch Syndrome (**Fig 4**). Cancer genes are enriched in the SSM-prone genes (**Fig 3A**, red category). Cancer genes in this category have higher predicted haploinsufficiency than cancer genes associated with lower levels of SSMs (**Fig 4D**). Machine learning was used to determine other features associated with the SSM-prone genes (**Fig 5A**). In general, no single feature dominated, rather a combination of features determined whether a disease gene was prone to splicing mutations. However, there are certain properties of splicing mutation that warrant further consideration. Splicing disruptions are potent loss of function mutations. This property probably explains the evidence of haploinsufficiency in the SSM-prone genes. Finally, unlike protein coding variants, splicing variants could have tissue specific affects. Consistent with a model of tissue specific affects, Lynch syndrome causes a wide variety of cancer types. While beyond the scope of this work, further studies will be needed to explore tissue specific differences in splicing for Lynch syndrome mutations.

As there is a high medical importance in discovering new cancer genes, the random forest classifier that was trained on the set of 86 SSM-prone genes was applied across the entire genome to reveal a set of 499 predicted SSM-prone genes. One possibility is these 499 SSM-prone genes could be targets of splicing factors that contain dominant oncogenic mutations (e.g. SF3B1, U2AF1) [33–35]. Highly significant enrichment in the overlap between the targets of these driver mutations and SSM-prone genes was observed. However, this enrichment disappeared when a correction for intron number was applied to the analysis. While little is known about this novel set of genes, the mark of purifying selection is evident in the degree of variation tolerated in these genes. Using the ExAC dataset, significantly fewer variants are tolerated within splice site regions in the predicted SSM-prone genes. Stratifying these variants by the degree to which the mutation disrupts the splice site suggests a strong selection against splicing mutations in common SNPs. In other words, variants that significantly decrease the PWM scores at the 5′ ss and 3′ ss are underrepresented in common SNPs implying that they are removed by natural selection before they reach MAF >0.01 in the human population (**Fig 5E, S4 Fig**). The finding that more than half of the 499 predicted SSM-prone genes also do not tolerate premature stop codons is further indication of strong selection (**S5 Fig**). While it is beyond the scope of this work to define the role and function of each of these genes, there is an indication that many relate to cancer. Of the 12 GO terms enriched in this set, 4 categories were also associated with the original set of cancer genes suggesting the existence of novel cancer genes (comparison of COSMIC cancer gene GO terms and 499 predicted SSM-prone gene GO terms). Taken together these findings suggest a set of genes that should be prioritized in the analysis of clinical sequencing data with a particular emphasis on cancer.

## Materials and methods

### Splicing efficiency analyses of exonic mutations

The 36 exonic *MLH1* mutations assessed for splicing defects mapped to internal exons and were selected based on their classification of being disease causing (DM) with a previously undocumented role in splicing. The splicing efficiency of wildtype and mutant exons was calculated as below:

$${log}_{2}\left(\frac{{spl}_{i}∕\sum_{i=1}^{n}spl}{{inp}_{i}∕\sum_{i=1}^{n}inp}\right)$$

where *spl_i_* is the count for spliced output *i,inp_i_* is the count for input *i*, and *n* is the number of species that were analyzed in the library pool. MaPSy experiments in vivo and in vitro were performed as previously described [10]. Briefly, solid-phase oligonucleotide synthesis technology was used to generate a 200 nt fragment (200-mer) that included both the wildtype and mutant exons, 15 nt of the downstream intron and ≥55 nt of the upstream intron, and were flanked by 15-mer common primer sequences.

The *in vivo* splicing reporters were generated using overlapping PCR and consists of the Cytomegalovirus (CMV) promotor, Adenovirus (pHMS81) exon with part of its downstream intron at the 5′ end, followed by the 200-mer library, and exon 16 of *ACTN1* with part of intron 15 and the bGH polyA signal sequence at the 3′ end. The resulting *in vivo* reporters were transfected into human embryonic kidney hek293T cells. After 24 hours of transfection, RNA was extracted and both the input reporters and spliced species were sequenced.

The *in vitro* splicing reporters have a similar design to the in vivo reporters, but exclude the ACTN1 exon, and the CMV promoter was replaced with the T7 promoter. The *in vitro* splicing reporters were obtained through in vitro transcription using T7 RNA Polymerase. The resulting RNA was then used for splicing reactions in 40% HeLa-S3 nuclear extract. Pools of the input and spliced RNAs were converted to cDNA and prepped for deep sequencing.

The allele ratios between wildtype and mutant exons in the different spliceosomal fractions were obtained as follows:

$${log}_{2}\left(\frac{{mi}_{e}/{mi}_{i}}{{mj}_{e}/{mj}_{i}}\right)$$

where *mi_e_* and *mi_i_* is the counts for the minor allele in the selected pool and input, respectively, *mj_e_* and *mj_i_* is the counts for the major allele in the selected pool and input, respectively. For each wildtype-mutant pair, the allele that splices more efficiently is assigned as the major allele.

### Validation

Wildtype and mutant sequences of exon 15 of MLH1 (NM_000249.3:c.1684C-T), exon 2 of BRCA1 (NM_007294.3:c.5425G-T) and exon 12 of OPA1 (NM_015560.2:c.1199C-T) were synthesized by Synbio Tech (Monmouth Junction, NJ) and incorporated into MaPSy in vivo backbone (Adenovirus (HMS81) and ACTN1 exon 15 by overlapping PCR [10]. MaPSy constructs were transfected into 293T cells and RNA were extracted after 24 hours. RT-PCR were subsequently performed and ran on 1.5% agarose gel, as previously described [10].

### ESR mapping

Hexamer ESEs and ESSs were downloaded from published data (17). A sliding window of 1 nucleotide was used plot the predicted ESEs and ESSs in the *MLH1* exons assayed with MaPSy (**S1 Fig**). The ‘ESR wt/mt difference’ in **S1 Table** was computed as the wild type-mutant difference in hexamer scores (17).

### Simulations

Disease causing splicing and coding sequence mutations (DM–disease mutations) were selected from the Human Genome Mutation Database (HGMD). The mutations were then classified as SSM, missense, or nonsense mutations. To be considered an SSM, the variant was required to be within the canonical splice-sites (-3 to +6 positions at the 5′ ss and -20 to +3 at the 3′ ss) and labeled as a splicing mutation by HGMD. The number of missense, nonsense, and SSM mutations were determined for each intron-containing gene.

#### Scatter plot of total mutations vs. splicing mutations

The total number of mutations were plotted against the total number of SSM in a gene. Weighted random sampling was then used to construct a 99.9% confidence interval that capitulates the expected number of SSM given the total number of mutations within a gene. Using the proportion of total SSM to total mutations in the HGMD as a weight for random sampling (**Eq 1**), the proportion of SSM given the total mutations in each gene was simulated 1,000 times. Genes falling outside the simulated values represent genes that have more (above the confidence interval) or less (below the confidence interval) SSM than expected (p-value <0.01) based on the distribution of mutations types within the dataset.

$$weight=\left(\frac{{total}_{spl}}{{total}_{muts}}\right)$$ (Eq 1)

#### Normalized simulation

The ratio in the previous simulation was normalized to the total number of splice sites (ss) and the total length of the coding sequence (CDS) in the HGMD dataset (**Eq 2**). To obtain a weight for random sampling using this ratio, the number of SS and the length of the CDS for each gene was used to obtain a unique SSM to total mutations weight for each gene (**Eq 3**). The corresponding weights were then used as before to simulate 1,000 times the expected number of SSM for each gene. Genes that have more or less SSM than the simulated values have more or less SSM than expected, respectively.

$${Normalized}_{weight}=(\frac{{total}_{spl}}{{total}_{muts}})(\frac{{total}_{CDS}}{{total}_{SS}})$$ (Eq 2)

$${Gene}_{weight}=(\frac{{total}_{spl}}{{total}_{muts}})(\frac{{total}_{CDS}}{{total}_{SS}})(\frac{{gene}_{SS}}{{gene}_{CDS}})$$ (Eq 3)

#### ESM exon simulation

The total number of mutations tested using MaPSy per exon were plotted against the total number of mutations that altered splicing (ESM). Weighted random sampling was then used to construct a 99.9% confidence interval that capitulates the expected number of ESM given the total number of mutations tested in an exon. Using the proportion of total ESM to total mutations in the an exon as a weight for random sampling (**Eq 4**), the proportion of ESM given the total mutations in each exon was simulated 1,000 times. Exons falling outside the simulated values represent genes that have more (above the confidence interval) ESM than expected (p-value <0.01) based on the total number of mutations tested using MaPSy and the total number of ESMs identified using MaPSy.

$$weight=\left(\frac{{total}_{ESM}}{{total}_{muts\;tested}}\right)$$ (Eq 4)

### Gene Ontology (GO) enrichment analysis

The list of 86 SSM-prone genes from HGMD and the list of 499 predicted SSM-prone genes were analyzed for the enrichment of specific GO terms using the PANTHER GO-Slim Biological Process annotation data set provided by the PANTHER Classification System.

### Enrichment of cancer genes in HGMD SSM predisposed genes

The list of cancer genes provided by the Catalogue of Somatic Mutations in Cancer (COSMIC) was downloaded and intersected with the list of HGMD genes. A permutation test was then performed to determine if cancer genes were overrepresented in the SSM-prone genes.

### Random forest predictor variables and features

ESS, ESE, and ESR’s were downloaded from published data [17] and the density was calculated by dividing the total number of regulatory elements by the length of the exonic sequences and averaging the density per gene. SNP density was calculated using the list of common SNPs (MAF > 0.01) provided by exome consortium [26] and dividing by the length of the exonic sequence (‘Exon SNP dens’) or the length of the gene (‘Gene SNP dens’). Conservation was scored using PhastCons46way placental for both the exonic sequences (‘Exon Cons’) and coding sequence (‘Gene Cons’). The free energy estimate (∆G) was computed using RNAfold [27], with default settings for both the exonic sequences (‘Exon ∆G’) and the for 70 nucleotides up- and down-stream of the splice-sites (‘SS ∆G’). Haploinsufficiency scores were obtained from a previous study that developed a haploinsufficiency prediction model using a large deletion data set (Wellcome Trust Consortium) [23]. Splice site strength was calculated using perl scripts from the MaxEntScan [28]. ExAC variant conservation was determined using the intersection of the ‘phastCons100way’ track with ‘ExAC Variant’ locations over each gene reported in the HGMD. The intersection generated an average conservation score for the variant sites in each gene based on a zero to one scale.

### Random forest classification and measure of variable importance

R implementation of random forest, package ‘randomForest’ [29], was used to determine the individual contribution of various functional genomic features (see ‘Random forest predictor variables and features’ methods section) in distinguishing SSM-prone genes from non-SSM-prone genes and to generate a predictive model. ‘randomForest’ is a nonparametric ensemble learning method where individual trees (*k*th trees) in a forest are constructed based off a different sub-sample (bootstrap sample) from the original training set and then averaged to provide unbiased estimates of predicted values. Two-thirds of the training set was used for the construction of the *k*th trees with the remaining one-third (out-of-bag data) used for cross-validation and estimates of variable importance. Default parameters were used to construct the random forest model, with the exception that ‘strata’ was used to sample the majority class (genes with the expected number of SSM) to make the frequency of the expected class closer to the frequency of the rarest class (genes with more SSM than expected). Variable importance was measured by the degree of model accuracy decrease with the permutation of a single predictor variable. The larger the mean decease in accuracy, the more important the variable is deemed in the classification of the data.

### Logistic regression classification

R implementation of logistic regression, ‘glm()’ function, was used to generate a predictive model for distinguishing SSM-prone genes from non-SSM-prone genes. Logistic regression is a classification method that relies on fitting a regression curve given a set of predictor variables and categorical response variables. Again, two-thirds of the data was used to construct the model with the remaining one-third of the data used for cross-validation. Default parameters were used to construct the logistic regression model, with the exception that ‘family = ‘ was set to binomial.

### Predicting novel SSM-prone genes

The random forest model generated from the HGMD dataset was then applied to the rest of the testable genes in genome. Testable genes were required to be void of a previously described disease phenotype by HGMD, contain introns, and have sufficient genomic feature data. This resulted in ~13,000 genes that could be tested using the random forest predictive model. The ‘predict()’ function with ‘type = ‘ set to ‘prob’ was used to predict SSM-prone genes based on a probability estimate. A probability threshold of > 0.6 was set to select SSM-prone genes, which resulted in 499 predicted SSM-prone gene.

### Splice site ExAC variation in predicted SSM-prone genes

All low frequency (MAF < 0.01%) single nucleotide ExAC variants reported in the splice site regions of genes (-3 to +6 position at the 5′ ss and -20 to +3 position at the 3′ ss) were counted for each gene and divided by the number of SS’s. The list of ExAC SS region variants per SS was then intersected with the genomic genes tested using the random forest model. The genes were then divided into genes predicted to be SSM-prone (*n* = 497, after intersection) and genes predicted with a high probability (prob > 0.6) to have the expected number of SSM (*n* = 5995). The average ExAC SS region variants per splice site were plotted for genes predicted to be prone SSM and genes with the expected number of SSM.

### Evaluation of selective pressure on splice site signal

The 499 predicted SSM-prone genes were intersected with RefSeq database and only the ones having RefSeq transcript id were retained for the downstream analysis (*n* = 486). All ExAC variants that fall within the splice sites (both 3′ and 5′) of the 486 genes were scored using the Maximum entropy model for splice sites (PMID: 15285897). The ExAC variants were separated based on their minor allele frequency into rare (MAF < 0.01%) and common (MAF > 1%). The entire distribution of scores and the two classes of alleles were plotted. The collapsed plots based on splice site score threshold were also generated.

### ExAC’s PTV-intolerant genes and predicted SSM-prone gene comparison

The list of 3,230 genes depleted of predicted PTV’s in ExAC (PTV-intolerant) were intersected with the list of genomic genes analyzed with the random forest model. 1,746 PTV-intolerant genes were analyzed using the random forest model. 281 of 1,746 were predicted to be prone to SSM. The intersection of the two datasets was plotted as a Venn diagram and significance was determined using the Fischer’s exact test.

## Supporting information

S1 Fig Map of predicted ESR’s in *MLH1* exons analyzed in MaPSy. Predicted ESE’s (bottom brackets) and ESS’s (upper brackets) [17] were mapped to the *MLH1* exons analyzed with MaPSy. Positions of exonic mutations assayed are highlighted in blue (no effect on splicing) and red (resulting in defective splicing). Positions that had more than one mapped mutation are bold. The sequences for both the branch point sequence (BP Seq) and polypyrimidine tract (PY Tract) are also noted.
(TIF)

S2 Fig Exons enriched for ESMs. ESM versus all HGMD exonic mutations tested in MaPSy exons with regions of 99.9% confidence interval shown in gray. **B.** List of exons enriched for ESMs (*P* < 0.01).
(TIF)

S3 Fig Example MaPSy assembly assay and validation in patient samples. **A**. The results from RT-PCR of the output RNA (spliced species) from MaPSy for three replicates is shown. **B.** Spliceosomal complexes (B/C, A, E) visualized in native gels for the MaPSy heterogeneous library substrates. **C**. Migration of RNA splicing intermediates from MaPSy heterogeneous library substrates.
(TIF)

S4 Fig TSG are prone to splicing dysfunction. Average percent SSM and ESM in COSMIC identified oncogenes vs non-oncogenes and TSG vs non-TSG listed in HGMD. Star indicates a significant difference between gene groups (*P* < 0.01, Mann-Whitney U test).
(TIF)

S5 Fig Sample genomic features associated with SSM-prone genes. Average number of introns, exon length, SS ∆G, HI score, and ExAC variant conservation score in genes with more SSM than expected (Upper, red bar), expected SSM (Expected, blue bar), and less SSM than expected (Lower, green bar). *P-*values calculated using Kruskall-Wallis test.
(TIF)

S6 Fig Enrichment of rare ExAC variants in the functional 3′ splice-site signal category. **A.** Common variants are depleted from the category of variants that cause loss of splice site signal at the 3′ splice site. **B.** Rare variants are enriched in the range of the splice site signal scores that abolish 3′ splice site recognition.
(TIF)

S7 Fig Overlap of ExAC’s PTV-intolerant genes and novel 499 predicted SSM-prone genes. Enrichment of ExAC’s PTV-intolerant genes in the 499 genomic genes predicted to be susceptible to SSM (*P* = 7.53e-98, Fisher Exact).
(TIF)

S1 Table Variants in MLH1 analyzed with MaPSy. (XLS)

S2 Table HGMD SSM-prone genes. (XLS)

S3 Table GO term enrichment analysis of 86 SSM-prone genes. (PDF)

S4 Table Features used in machine learning. (PDF)

S5 Table HGMD SSM-prone genes based on normalized simulation. (XLS)

S6 Table Cross-validation of random forest. (XLSX)

S7 Table 499 predicted SSM-prone genes, PTV intolerance, and individual GO term associations. (XLS)

S8 Table Go Term enrichment analysis of the 499 predicted SSM-prone genes. (PDF)

S9 Table SSM-prone cancer genes with ESM browser links. (XLSX)
