# Supplementary figures and images for: Hereditary cancer genes are highly susceptible to splicing mutations
Source: PLoS Genet. 2018 Mar 5;14(3):e1007231. doi: 10.1371/journal.pgen.1007231 (PMC5854443; doi:10.1371/journal.pgen.1007231)

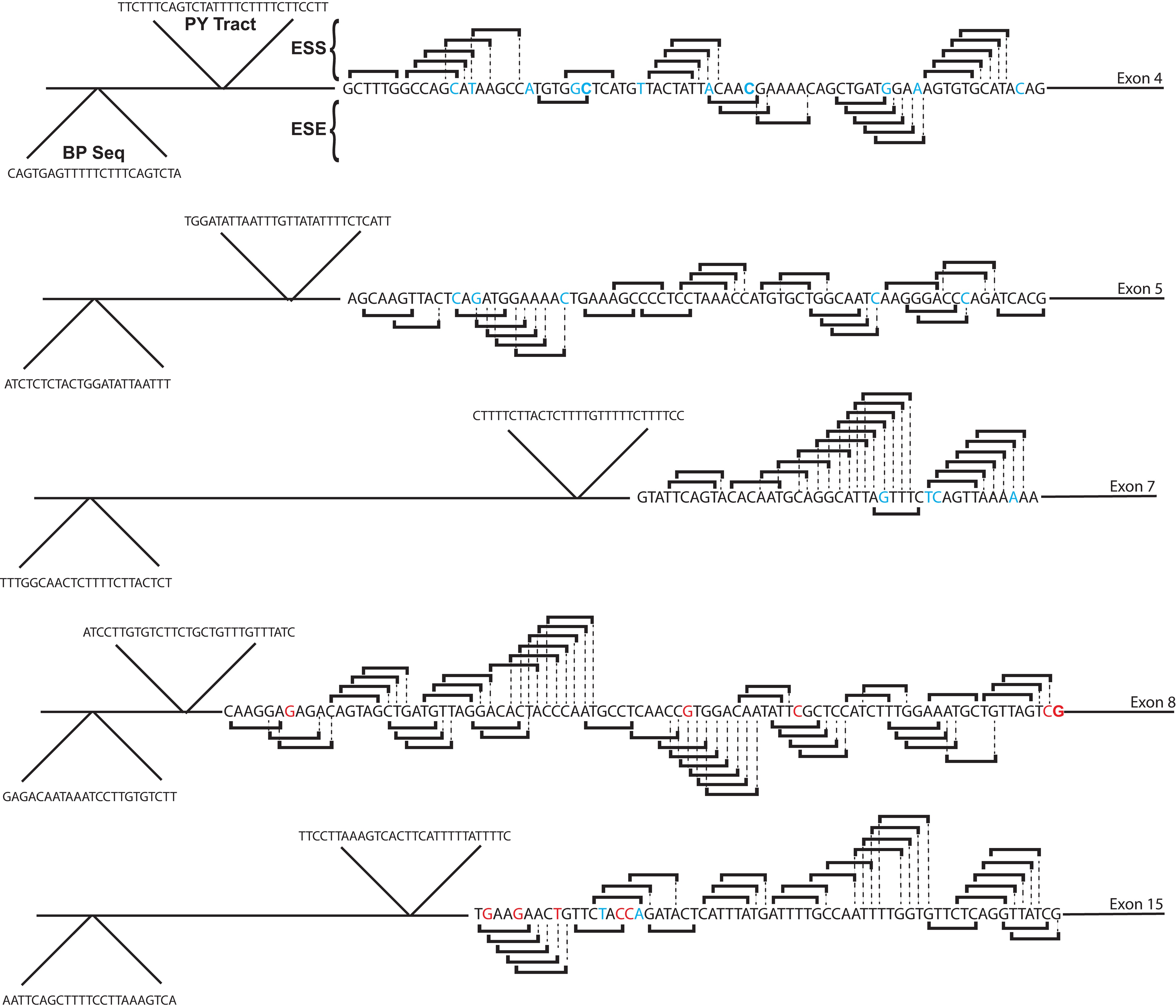

Supplement: S1 Fig — Predicted ESE’s (bottom brackets) and ESS’s (upper brackets) [17] were mapped to the MLH1 exons analyzed with MaPSy. Positions of exonic mutations assayed are highlighted in blue (no effect on splicing) and red (resulting in defective splicing). Positions that had more than one mapped mutation are bold. The sequences for both the branch point sequence (BP Seq) and polypyrimidine tract (PY Tract) are also noted. (TIF) [file pgen.1007231.s001.tif]

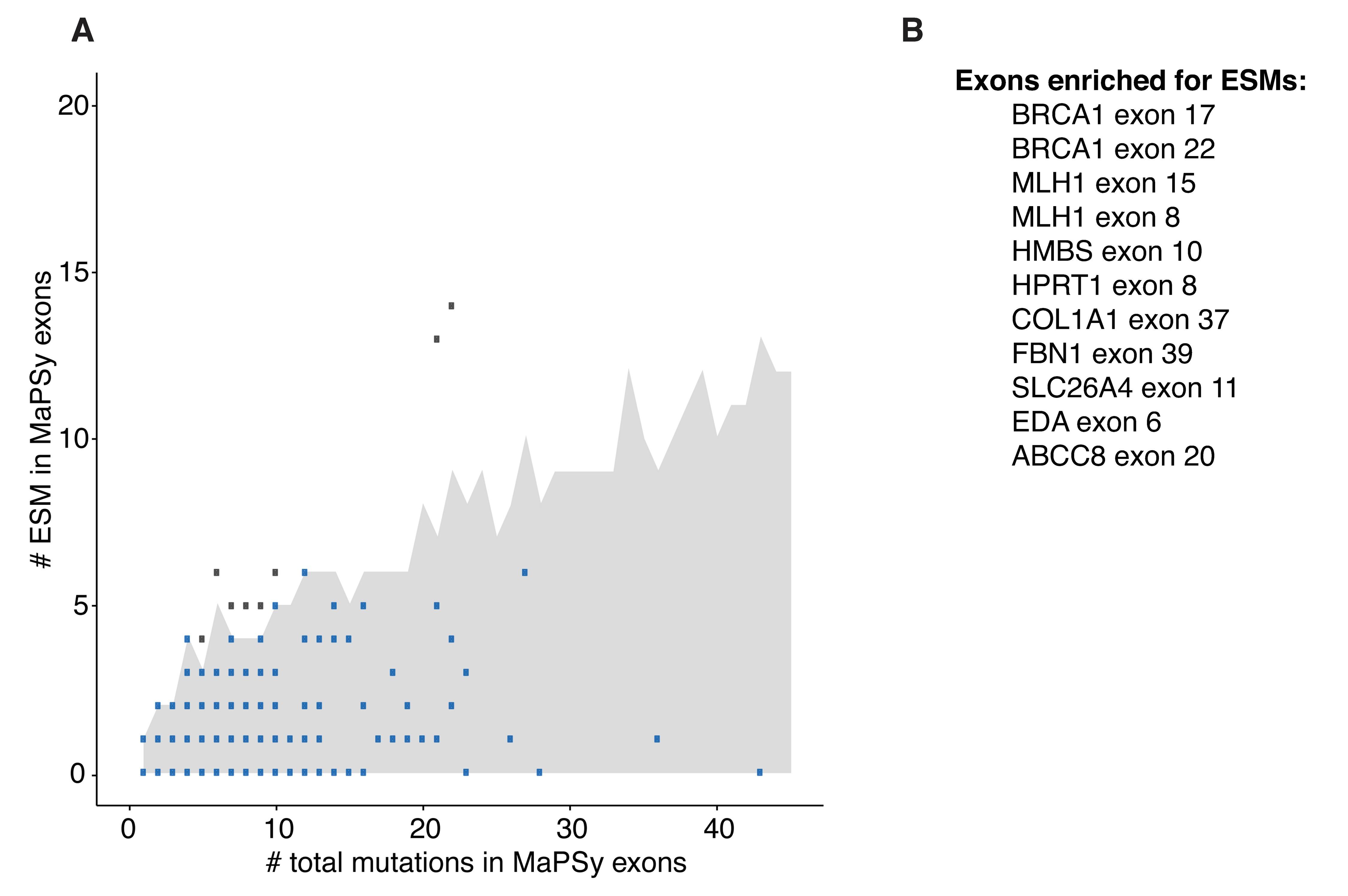

Supplement: S2 Fig — ESM versus all HGMD exonic mutations tested in MaPSy exons with regions of 99.9% confidence interval shown in gray. B. List of exons enriched for ESMs (P < 0.01). (TIF) [file pgen.1007231.s002.tif]

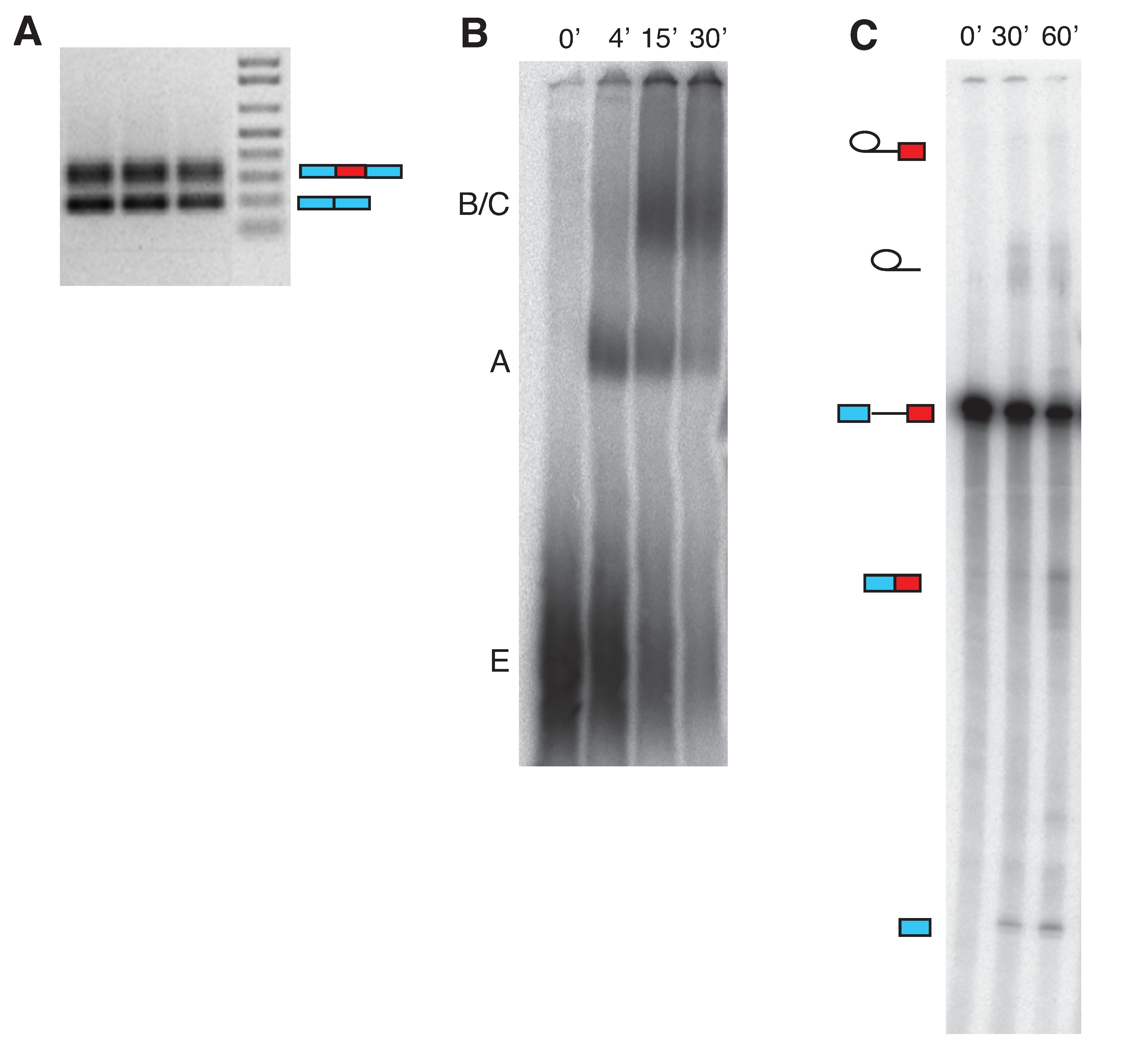

Supplement: S3 Fig — A. The results from RT-PCR of the output RNA (spliced species) from MaPSy for three replicates is shown. B. Spliceosomal complexes (B/C, A, E) visualized in native gels for the MaPSy heterogeneous library substrates. C. Migration of RNA splicing intermediates from MaPSy heterogeneous library substrates. (TIF) [file pgen.1007231.s003.tif]

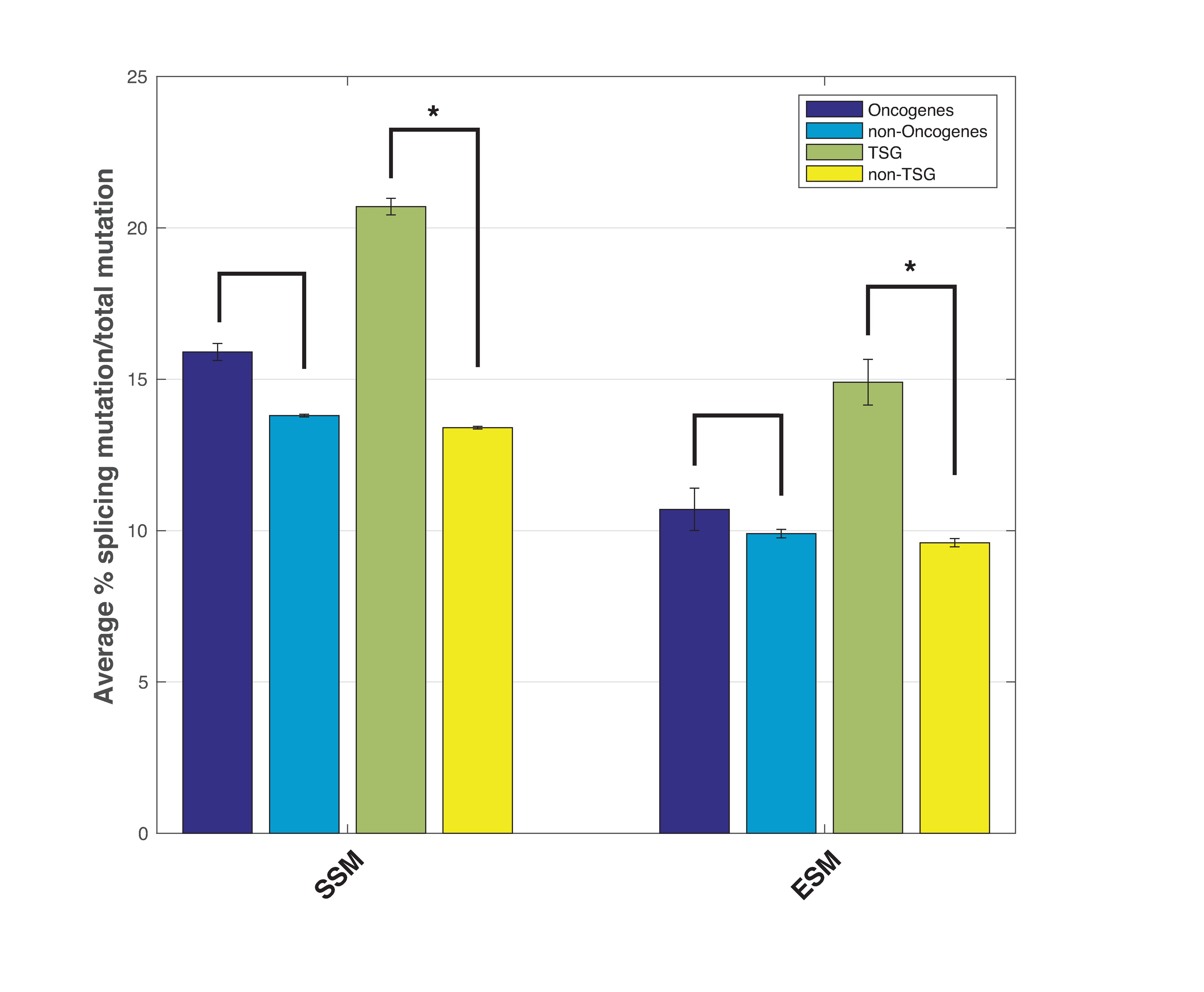

Supplement: S4 Fig — Average percent SSM and ESM in COSMIC identified oncogenes vs non-oncogenes and TSG vs non-TSG listed in HGMD. Star indicates a significant difference between gene groups (P < 0.01, Mann-Whitney U test). (TIF) [file pgen.1007231.s004.tif]

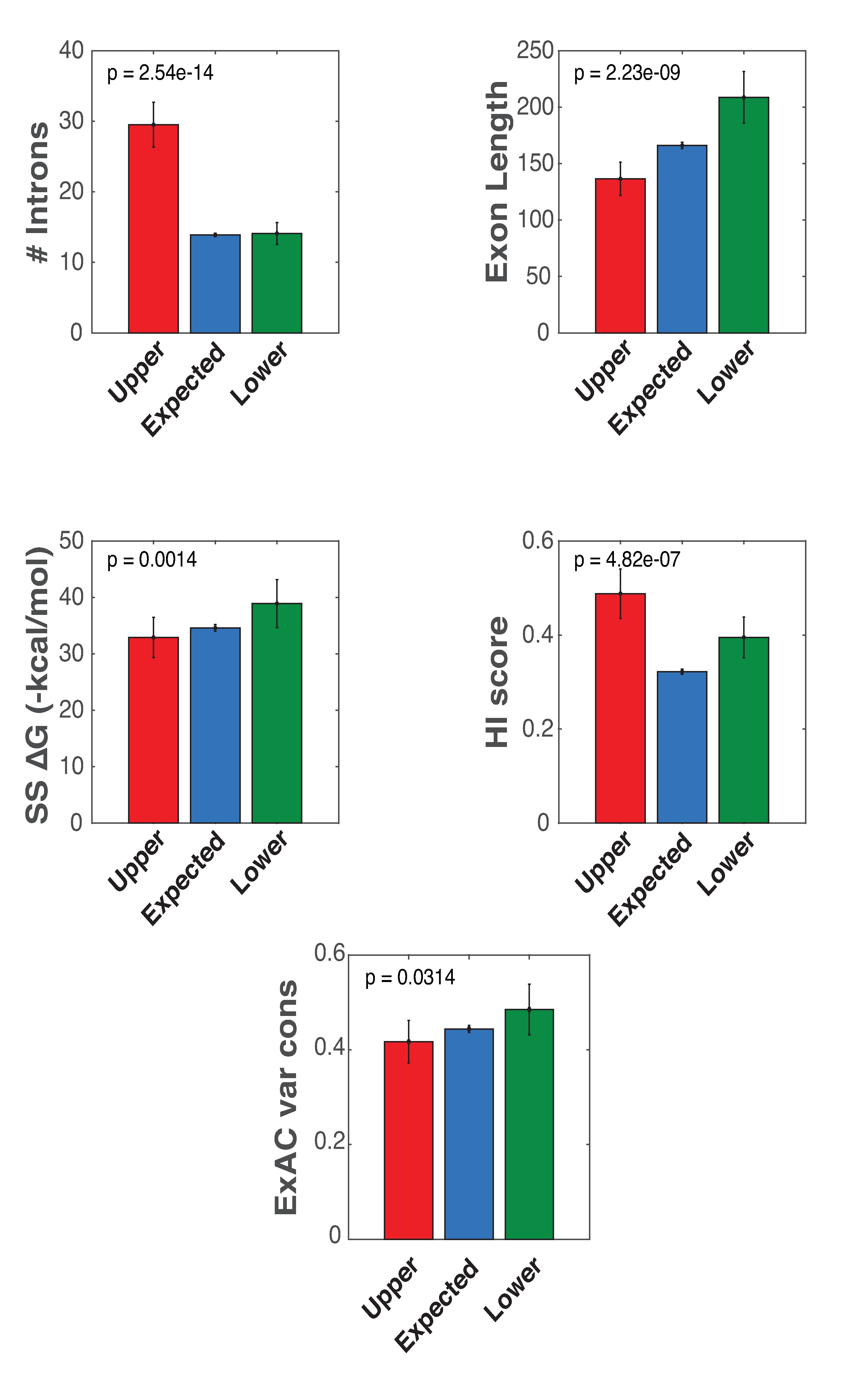

Supplement: S5 Fig — Average number of introns, exon length, SS ∆G, HI score, and ExAC variant conservation score in genes with more SSM than expected (Upper, red bar), expected SSM (Expected, blue bar), and less SSM than expected (Lower, green bar). P-values calculated using Kruskall-Wallis test. (TIF) [file pgen.1007231.s005.tif]

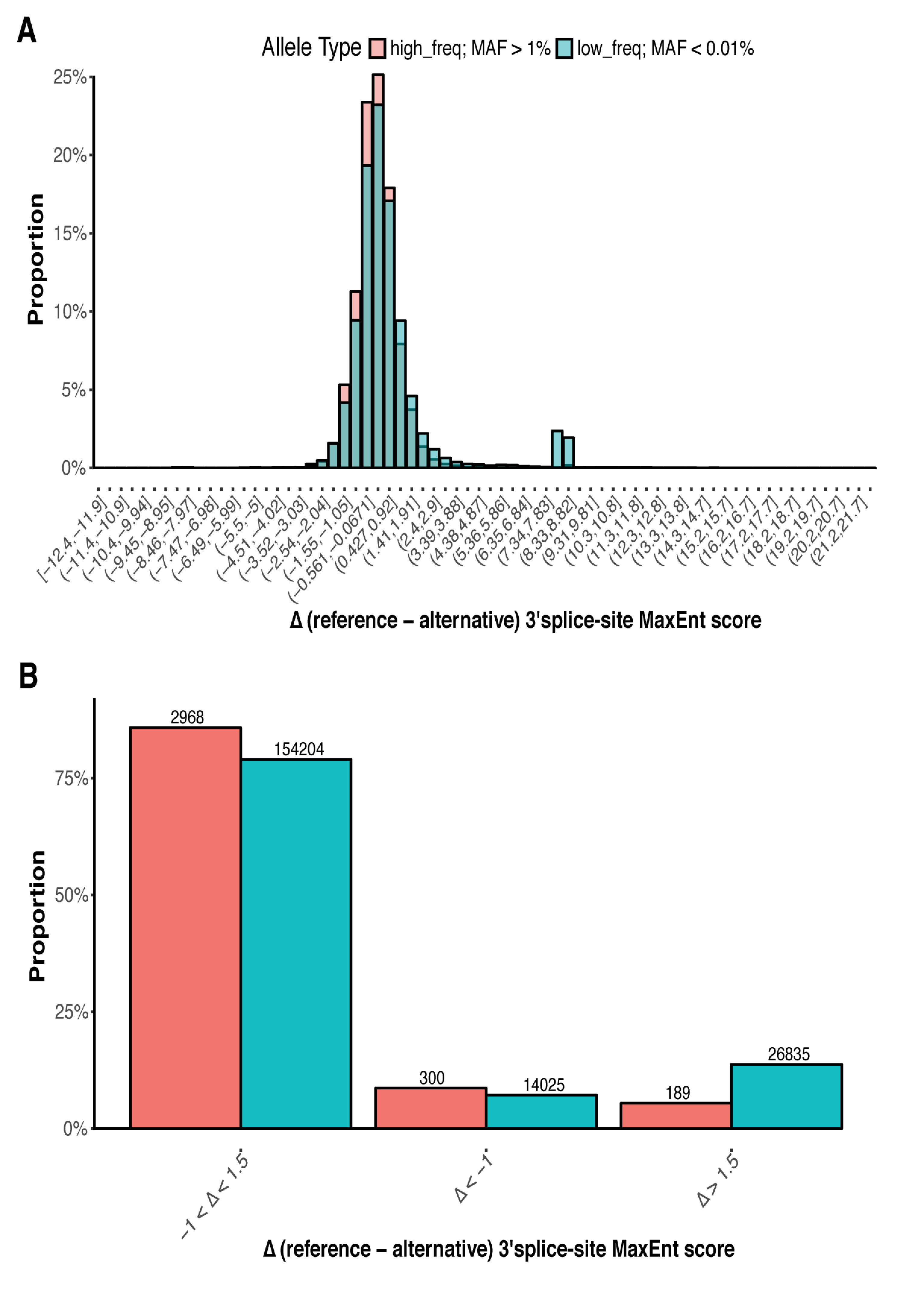

Supplement: S6 Fig — A. Common variants are depleted from the category of variants that cause loss of splice site signal at the 3′ splice site. B. Rare variants are enriched in the range of the splice site signal scores that abolish 3′ splice site recognition. (TIF) [file pgen.1007231.s006.tif]

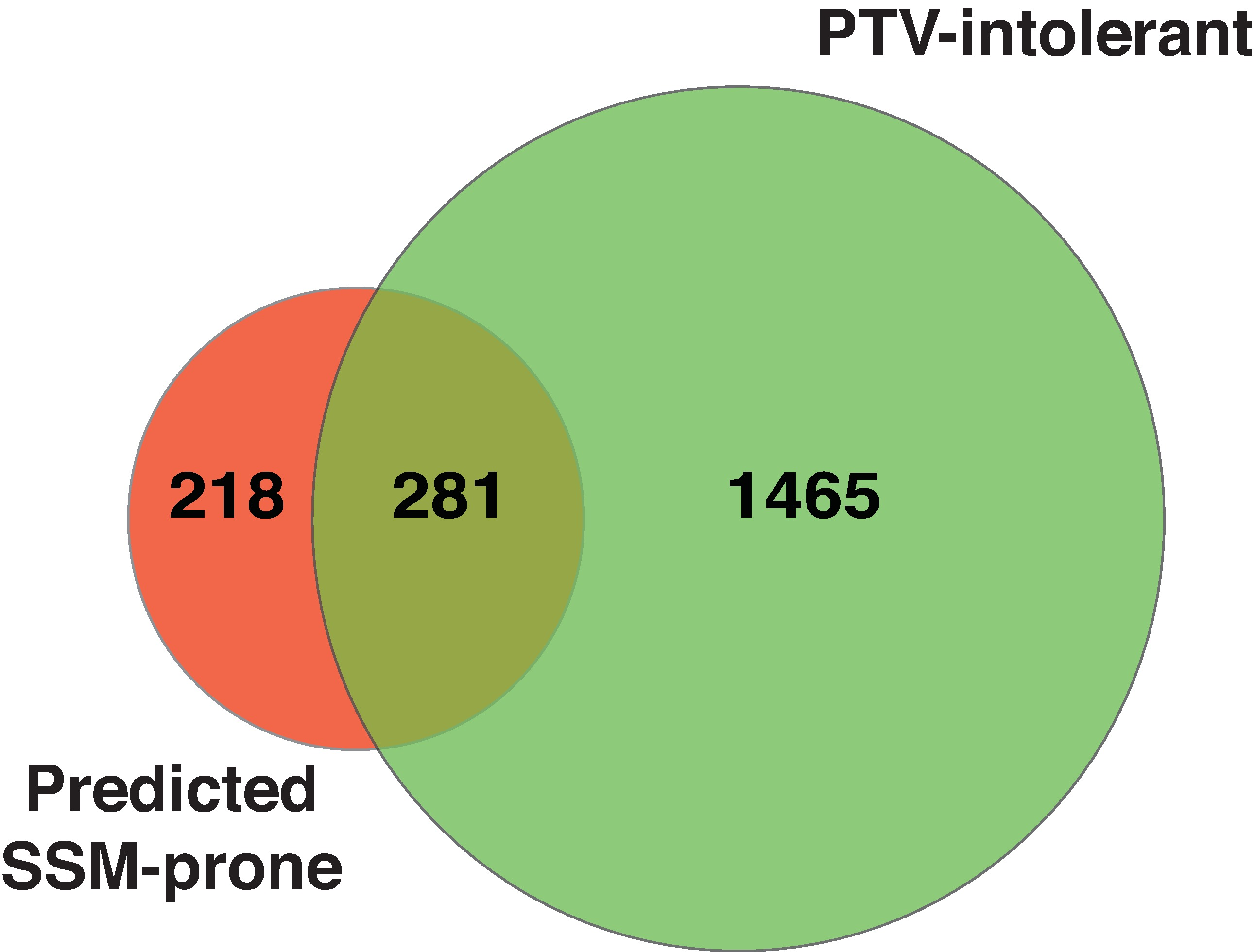

Supplement: S7 Fig — Enrichment of ExAC’s PTV-intolerant genes in the 499 genomic genes predicted to be susceptible to SSM (P = 7.53e-98, Fisher Exact). (TIF) [file pgen.1007231.s007.tif]
