# Supplementary material for: Hereditary cancer genes are highly susceptible to splicing mutations
Source: PLoS Genet. 2018 Mar 5;14(3):e1007231. doi: 10.1371/journal.pgen.1007231 (PMC5854443; doi:10.1371/journal.pgen.1007231)
Supplement: S3 Table — (PDF) [file pgen.1007231.s010.pdf]

**Biological Process**	***P*-Value**
Macrophage Activation	3.33E-03
Blood Ciruclation	8.22E-03
DNA repair	2.70E-02
Anatomical Structure Morphogenesis	2.70E-02
