# Supplementary material for: Hereditary cancer genes are highly susceptible to splicing mutations
Source: PLoS Genet. 2018 Mar 5;14(3):e1007231. doi: 10.1371/journal.pgen.1007231 (PMC5854443; doi:10.1371/journal.pgen.1007231)
Supplement: S4 Table — (PDF) [file pgen.1007231.s011.pdf]

Feature	Resource
ESS Density	Chasin ESSseqs (23)
ESE Density	Chasin ESEseqs (23)
ESR Density	Chasin ESRseqs (23)
Gene SNP Density	Density of Exome Consortium SNPs (AF > 0.01) (24)
Exon SNP density	Density of Exome Consortium SNPs (AF > 0.01) (24)
Exon Conservation	PhastCons46wayPlacental
Gene Conservation	PhastCons46wayPlacental
Exon ΔG	RNAfold (25)
HI	Haploinsufficiency Score (20)
SS ΔG	RNAfold (25)
3'ss score	MaxEntScan (26)
5'ss score	MaxEntScan (26)
Exon Length	--
Intron Length	--
CDS length	--
Exon GC content	--
Intron GC	--
GC differential	--
Number of introns	--
