# Supplementary material for: Hereditary cancer genes are highly susceptible to splicing mutations
Source: PLoS Genet. 2018 Mar 5;14(3):e1007231. doi: 10.1371/journal.pgen.1007231 (PMC5854443; doi:10.1371/journal.pgen.1007231)
Supplement: S8 Table — (PDF) [file pgen.1007231.s015.pdf]

**Biological Process**	***P*-Value**
Nuclear Transport	1.52E-11
Intracellular Protein Transport	6.92E-11
Catabolic Process	3.02E-08
Phosphate-containing Compound Metabolic Process	1.62E-06
Protein Localization	2.53E-06
*Mitosis	5.08E-05
Nitrogen Compound Metabolic Process	2.07E-03
Nucleobase-containing Compound Metabolic Process	2.52E-03
Cellular Component Morphogenesis	2.10E-02
*Regulation of Cell Cycle	2.20E-02
Exocytosis	2.99E-02
Cellular Protein Modification	4.27E-02
